# A lean-six sigma–ergonomics index for assessing shop-floor operational resilience readiness: Method development and SME demonstration

**DOI:** 10.1016/j.mex.2026.104188

**Published:** 2026-10-02

**Authors:** Deri Maryadi, Moses Laksono Singgih, Dyah Santhi Dewi

**Affiliations:** a Department of Industrial and Systems Engineering, Institut Teknologi Sepuluh Nopember (ITS), Surabaya, 60111, Indonesia; b Department of Industrial Engineering, Universitas Tridinanti, Palembang, 30129, Indonesia

**Keywords:** Operational resilience index, Lean six sigma, Ergonomics, Manufacturing SMEs, Shop-floor assessment, Human-system resilience

## Abstract

Operational resilience readiness at the shop-floor level depends on efficient process flow, stable quality, and adequate human–system capacity, yet these dimensions are commonly assessed separately. This article presents the Lean-Six Sigma–Ergonomics Operational Resilience Index (LSE–ORI), a non-interventional composite method for assessing manufacturing conditions that support resilience readiness. Process-flow efficiency is represented by Process Cycle Efficiency, quality stability by the actual sigma level normalized against a defined target, and human–system capacity by physical, cognitive, and macroergonomic assessments. The scores are standardized to a 0–1 scale and combined multiplicatively to produce the index and identify the lowest-scoring dimension. A two-month demonstration involving 13 operators on a tractor spare-parts production line yielded scores of 0.60, 0.54, and 0.62, resulting in an ORI of approximately 0.20. Quality stability was the lowest-scoring primary component, while work-system structural support was the weakest subcomponent within the human–system. Content validation by 15 experts across 64 items produced a minimum I-CVI of 0.933 and an S-CVI/Ave of 0.995, supporting applicability, calculation traceability, and content validity. Main advantages of the proposed method:

• Integrates three shop-floor dimensions into one assessment.

• Uses operational data without changing the production system.

Provides hierarchical diagnosis of the lowest-scoring component.

## Graphical abstract

**Figure:**
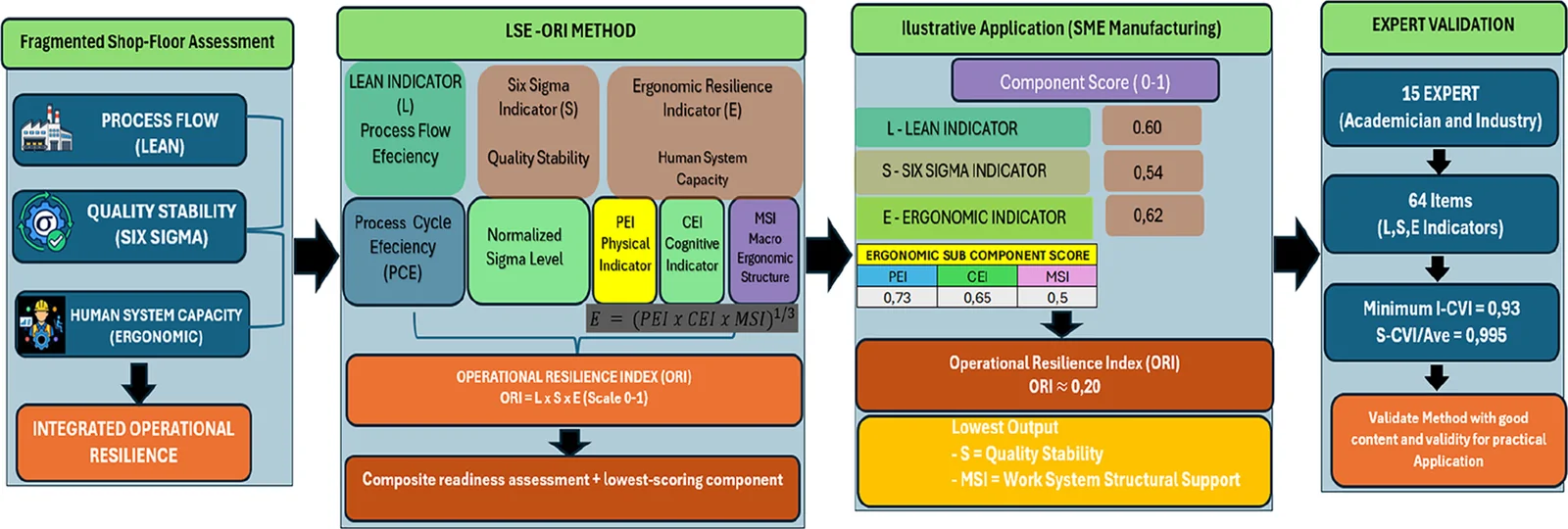

## Specifications table

**Table:** 

**Subject area**	Engineering
**Subject area**	Industrial and Manufacturing Engineering; Manufacturing Systems; Operational Resilience; Quality Engineering; Human Factors and Ergonomics
**More specific subject area**	Lean–Six Sigma–Ergonomics Operational Resilience Index Method (LSE–ORI Method)
**Name of your method**	Operational Resilience Index; Lean Six Sigma; Ergonomics; Manufacturing SMEs; Shop-floor assessment; Human-system resilience; Flexible measurement method
**Name and reference of original method**	It does not function as a single, original method. LSE–ORI is a new composite method that integrates established measures: Process Cycle Efficiency, normalized sigma level, RULA, REBA, NASA-TLX, and Macroergonomic Analysis of Structure. Original references for each constituent method are listed in the References section.
**Resource availability**	An LSE–ORI calculation worksheet, normalization procedures, anonymized SME demonstration data, a shop-floor data collection format, indicator implementation guidelines, an expert validation instrument, and content validity analysis results are provided as supplementary material. Additional operational data that cannot be published due to corporate confidentiality may be obtained from the corresponding author upon reasonable request and with the data owner's approval.

## Background

Operational resilience at the shop-floor level is becoming increasingly important, as manufacturing disruptions do not always manifest as major events that halt production [1,2]. Instead, operational vulnerabilities can arise from recurring micro-disruptions, such as waiting times [3], process flow imbalances [4], non-value-added activities [5], product defects [6], rework [7], postural work risk [8,9], mental workload [10,11], unclear procedures [12], and poor communication and coordination [[13], [14], [15]]. If not identified early, these conditions can compromise process continuity [16,17], quality stability [18], and the operators’ ability to maintain performance under operational pressure and adapt to disruptions [19,20]. These issues are particularly relevant to manufacturing systems that use manual or semi-manual production principles and still rely on operators' direct involvement.

Lean and Six Sigma have been widely employed to improve process flow and quality consistency, while ergonomics research has emphasized the importance of physical workload, cognitive demands, and work-system conditions in sustaining human performance [[21], [22], [23]]. In parallel, manufacturing-resilience studies have focused on organizational capabilities, system robustness, technological readiness, and recovery-oriented measures [[24], [25], [26], [27]]. Other studies have linked resilience engineering with ergonomic and macroergonomic considerations [28,29]. Despite these advances, these perspectives remain insufficiently integrated at the shop-floor measurement level. A traceable assessment method that jointly quantifies process-flow efficiency, quality stability, and human–system ergonomic capacity on a common measurement scale, while retaining component-level diagnostic information, remains insufficiently developed. Consequently, shop-floor vulnerabilities related to process flow, quality, and human–system conditions may still be evaluated separately, limiting the ability to determine which operational dimension most weakens resilience readiness. This unresolved measurement gap motivates the development of an integrated shop-floor assessment method.

Accordingly, this study aims to develop and demonstrate the Lean–Six Sigma–Ergonomics Operational Resilience Index (LSE–ORI) for assessing shop-floor operational resilience readiness. The method integrates three complementary dimensions: process-flow efficiency, represented by the Lean Indicator (L); quality stability, represented by the Six Sigma Indicator (S); and human–system ergonomic capacity, represented by the Ergonomic Resilience Indicator (E), comprising physical, cognitive, and macroergonomic components. The proposed method is demonstrated in a manufacturing SME and evaluated through expert content validation.

### Method overview and application scope

The Lean–Six Sigma–Ergonomics Operational Resilience Index Method (LSE–ORI Method) is a quantitative method for evaluating operational resilience at the manufacturing shop floor level. This method assesses a production system's internal capacity to maintain three complementary capabilities: smooth process flow, quality stability, and human-system ergonomic capacity. These three capabilities are represented sequentially by the Lean Indicator (L), Six Sigma Indicator (S), and Ergonomic Resilience Indicator (E). Their respective values ​​are normalized to a 0–1 scale and integrated to produce the Operational Resilience Index (ORI). The method is evaluative and observational; it does not require changes to layout, work methods, equipment, authority distribution, or production structure before measurement. Data are obtained under actual operating conditions through observation of process flows, quality recording, assessment of operator activities, measurement of cognitive workload, and evaluation of work system structures. Consequently, the LSE–ORI serves as an evaluative-preventive instrument for identifying sources of vulnerability before operational disruptions escalate into major production failures.

The unit of analysis may be a workstation, a production line, a group of interconnected processes, or a specific production flow with clearly defined start and end points. All measurements must use the same system boundary and observation period to ensure that the resulting component scores reflect a consistent operational setting. In the baseline configuration demonstrated in this article, process-flow efficiency is assessed using Process Cycle Efficiency, quality stability using the normalized actual sigma level, and human–system capacity through physical, cognitive, and macroergonomic assessments. The method may be adapted to different manufacturing settings by adjusting the assessment boundary, product or process characteristics, observation period, data sources, definitions of value-added activities and defect opportunities, and the physical ergonomics instrument. Any adjustment must preserve the underlying construct, produce a score that can be standardized to the common 0–1 scale, and maintain the interpretation that higher values indicate conditions that better support operational resilience readiness. Fig. 1 below summarizes the five-stage application procedure: defining the assessment scope, collecting the required shop-floor data, calculating the standardized component scores, deriving the composite index, and identifying the lowest-scoring component. The following subsection describes the indicator architecture and the relationship between the primary components and their constituent measures.

**Fig. 1:**
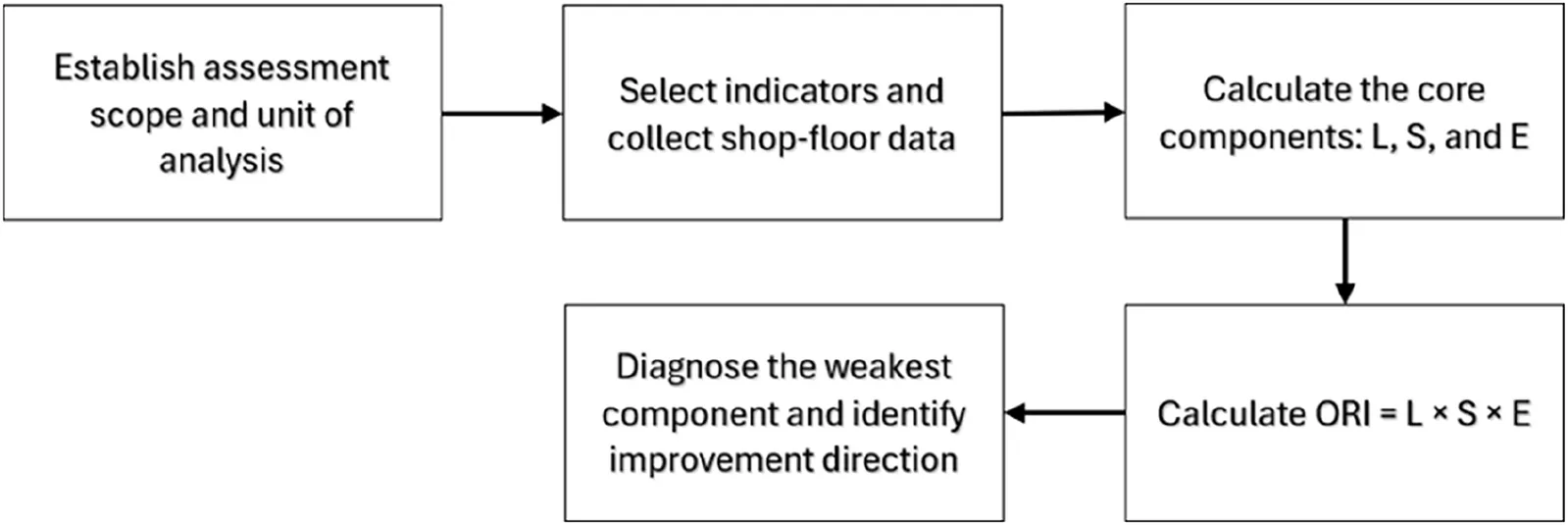
Overall procedure of the LSE–ORI method, from system-boundary definition and data collection to ORI calculation and weakest-component diagnosis.

### LSE–ORI architecture and indicator configuration

The LSE–ORI method is a hierarchical measurement structure that positions the Lean Indicator (L), Six Sigma Indicator (S), and Ergonomic Resilience Indicator (E) as the three primary components of operational resilience at the shop-floor level. These three components are integrated using a multiplicative formulation to derive the ORI value [30]. This formulation was selected because operational resilience requires the simultaneous support of process fluidity, quality stability, and human–system ergonomic capacity; consequently, a low value in any single component immediately lowers the ORI value and cannot be fully compensated for by high values in the other components [31,32].

The final stage compares the L, S, and E values to identify the component that contributes least to operational resilience. L assesses the fluidity of process flow, S assesses quality stability, and E assesses the capabilities of humans and work system structures in supporting operations. Thus, the LSE–ORI architecture decomposes operational resilience into traceable components without losing their integrative relationships.

The Lean Indicator and Six Sigma Indicator are operational components that directly assess process and quality conditions. In the basic configuration of this concept, L is represented by Process Cycle Efficiency, as this indicator reflects the proportion of value-added activity time relative to the total time required for the process flow. L is not intended to capture the full scope of Lean practices, but rather to provide a consistently calculable measure of production flow fluidity. S is represented by the actual sigma level normalized against the ideal target of six sigma. This component assesses the process's capability to consistently produce outputs that meet specifications. Therefore, L and S serve distinct functions: L relates to time structures and activity flows, whereas S relates to defects, variation, and quality stability. Details on the data, normalization, and formulas for both are provided in the measurement and calculation subsection to avoid repetition in the architecture discussion.

Component E possesses a multi-layered structure because the ergonomic capacity of human-system interaction cannot be adequately assessed solely based on the operator's physical condition. In this research configuration, E comprises the Physical Ergonomic Indicator (PEI), the Cognitive Ergonomic Indicator (CEI), and the Macroergonomic Structure Indicator (MSI). The PEI represents the operator's ability to perform activities without excessive postural risk, assessed using RULA and/or REBA depending on the nature of the activity [33,34]. The CEI represents the operator's capacity to handle mental demands, attention requirements, time pressure, and task complexity, as measured by NASA-TLX [35,36]. The MSI complements these two individual-level measures by evaluating the support provided by the work-system structure through Macroergonomic Analysis of Structure (MAS), which encompasses formalization, centralization, and complexity [37]. Formalization pertains to the clarity of procedures and work standards; centralization concerns the appropriate distribution of decision-making authority; and complexity concerns the manageability of interdepartmental relationships, communication, and coordination. Through this structure, E not only captures operator-level ergonomic conditions but also reflects whether the broader work system provides sufficient organizational support for coordinated and adaptive responses to operational pressures [38,39].

The LSE–ORI architecture distinguishes between the core constructs—which remain fixed—and the indicator configurations, which can be adjusted in a controlled manner, as summarized in Table 1. The constructs of process flow smoothness, quality stability, and human-system ergonomic capacity remain the foundation for L, S, and E. In the manufacturing SME demonstration configuration, PCE, normalized sigma level, RULA/REBA, NASA-TLX, and MAS are used because they suit manual or semi-manual production characteristics. Methodological flexibility applies mainly to selecting physical ergonomics instruments. RULA applies to activities predominantly involving the upper body, whereas REBA is better suited to tasks involving whole-body posture or manual material handling; both may be used concurrently if both types of risk are relevant. In different manufacturing contexts, alternative physical ergonomics instruments may be considered, provided they have a clear methodological basis, are convertible to a 0–1 scale and accurately represent physical ergonomics capacity. Such adjustments do not alter the roles of cognitive ergonomics and macroergonomics within the E component, nor do they change the fundamental L–S–E structure. Maintaining these boundaries is crucial to ensure that flexibility does not reduce LSE–ORI to a mere collection of interchangeable indicators that lose their underlying conceptual meaning.

**Table 1:** Components and basic indicator configuration of the LSE–ORI method.

Measurement level	Component	Construct represented	Basic indicator or instrument	Primary input data	Score processing	Final interpretation
Main component	Lean Indicator (L)	Process-flow efficiency	Process Cycle Efficiency (PCE) [40]	Value-added time and total lead time within the defined process boundary	PCE is calculated as the proportion of value-added time to total lead time and expressed on a 0–1 scale	A higher L value indicates a smoother production flow and a lower proportion of non-value-added time
Main component	Six Sigma Indicator (S)	Quality stability	Normalized actual sigma level [41]	Number of units produced, number of defects, number of defect opportunities per unit, DPO, DPMO, and actual sigma level	The actual sigma level is normalized against the defined target sigma level to obtain a 0–1 score	A higher S value indicates greater process-quality stability and a lower relative defect level
Ergonomic subcomponent	Physical Ergonomic Indicator (PEI)	Physical capacity of the human–work system	RULA and/or REBA [33,34]	Observed working posture, body movement, applied load, task frequency, and activity characteristics	RULA and/or REBA scores are reverse-normalized to a 0–1 scale so that a higher score represents a more favourable physical ergonomic condition	A higher PEI value indicates lower physical ergonomic risk and stronger physical support for operational resilience
Ergonomic subcomponent	Cognitive Ergonomic Indicator (CEI)	Cognitive capacity of operators	NASA Task Load Index (NASA-TLX) [36]	Mental demand, physical demand, temporal demand, performance, effort, and frustration	The overall NASA-TLX score is reverse-normalized from its original 0–100 scale to a 0–1 scale	A higher CEI value indicates a lower perceived workload and stronger cognitive capacity to support operational performance
Ergonomic subcomponent	Macroergonomic Structure Indicator (MSI)	Structural support provided by the work system	Macroergonomic Analysis of Structure (MAS) [29,42]	Assessment of formalization, centralization, complexity, communication, coordination, and decision authority	Item directions are aligned, dimension scores are normalized to a 0–1 scale, and the resulting scores are aggregated to form MSI	A higher MSI value indicates that the organizational and work-system structure provides stronger support for coordinated and adaptive operations
Ergonomic component	Ergonomic Resilience Indicator (E)	Overall human–system ergonomic capacity	Integrated PEI, CEI, and MSI scores	Normalized PEI, CEI, and MSI values obtained from the same unit and observation period	PEI, CEI, and MSI are integrated using geometric aggregation	A higher E value indicates stronger combined physical, cognitive, and macroergonomic support for operational resilience
Composite index	Operational Resilience Index (ORI)	Overall shop-floor operational resilience	Integrated L, S, and E scores	Lean Indicator, Six Sigma Indicator, and Ergonomic Resilience Indicator	The three main components are integrated using the multiplicative formulation (ORI = L × S × E)	A higher ORI value indicates stronger simultaneous support from process flow, quality stability, and human–system ergonomic capacity

### Component measurement and calculation

The calculation of the LSE–ORI begins by separately determining the values ​​for the Lean Indicator (L), Six Sigma Indicator (S), and Ergonomic Resilience Indicator (E). These three components use data from the same unit of analysis and observation period. All results are expressed on a scale of 0 to 1 with a consistent interpretation: higher values ​​indicate conditions that are increasingly supportive of operational resilience. This section describes only how each component is measured; the procedure for integrating the three is discussed in the subsequent subsection.

### Lean indicator

The Lean Indicator represents the smoothness of process flow within the observed production unit [3]. In the basic LSE–ORI configuration, the L value is calculated using Process Cycle Efficiency (PCE)—the ratio of the total time spent on value-added activities to the total time required for a product to pass through defined process boundaries [40]. This is determined through direct observation, time studies, process records, or a combination thereof. Value-added activities alter the form, function, or characteristics of a product to meet customer requirements. Meanwhile, total lead time encompasses the entire duration from when a product enters the initial observation boundary to when it exits the final boundary; this includes processing, waiting, material handling, inspection, and other non-value-added activities. The Lean Indicator is calculated using the following equation:

(1) $$L=\mathrm{PCE}=\frac{\mathrm{VAT}}{\mathrm{TLT}}$$

With: **L** = Lean Indicator value; **PCE** = Process Cycle Efficiency; **VAT** = value-added time, namely, the total time of activities that add value to the product; **TLT** = total lead time; this refers to the total time a product requires from the beginning to the end of the observed system boundaries. Since PCE is expressed as a ratio, the L-value falls directly within the 0–1 range and requires no additional normalization. An L-value approaching 1 indicates a higher proportion of value-added activities and suggests that the process flow is less affected by waiting times or non-value-added activities. Conversely, a low L-value indicates that delays, transfers, queuing, or other non-value-added activities consume a significant portion of process time. Within the LSE–ORI framework, PCE is not a standalone measure of operational resilience; rather, it represents the extent to which process-flow conditions support operational resilience at the shop-floor level.

### Six sigma indicator

The Six Sigma Indicator represents process quality stability [43]. The S-value is derived from the actual sigma level—based on production and defect data—and then compared against the ideal Six Sigma target [[44], [45], [46]]. The required data includes the number of units processed, the number of defects, the number of defect opportunities per unit, and quality inspection results. If the actual sigma level is not yet available in company records, the calculation can begin by determining defects per opportunity:

(2) $$\mathrm{DPO}=\frac{D}{N\;X\;O}$$

Where **DPO** = defects per opportunity; **D** = total number of defects found; **N** = number of product units inspected; **O** = the number of potential defect occurrences per unit. The DPO value is then converted into defects per million opportunities:

(3) DPMO=DPOX1.000.000

Where **DPMO** = number of defects per million opportunities, and **DPO** = number of defects per opportunity. The DPMO value is then converted to an actual sigma level using a conversion table or statistical software, and the conversion is applied consistently across all units of analysis. Once the actual sigma value is obtained, the Six Sigma Indicator is calculated as follows:

(4) $$S=\frac{\sigma \;\mathrm{Actual}}{\sigma \;\mathrm{Target}}\;So\;that,\;S=\frac{\sigma \;\mathrm{Actual}}{6\;\mathrm{sigma}}$$

With **S** = the Six Sigma Indicator value; ***σ actual*** = the actual sigma level derived from process defect data; ***σ target*** = the sigma level used as a benchmark; ***6 Sigma*** = the ideal Six Sigma target employed in the basic LSE–ORI configuration. An S-value close to 1 indicates that process capability is nearing the established quality target. Lower values ​​indicate a higher likelihood of defects and greater quality instability. Therefore, S is not interpreted as a raw sigma value, but rather as an index of quality stability achievement on a scale comparable to L and E. Data regarding production volume, defects, defect opportunities, DPMO, and defect rates are used solely to determine the actual sigma level; they are not treated as standalone ORI components.

### Ergonomic resilience indicator

The Ergonomic Resilience Indicator (E) represents the human-system's ergonomic capacity to support operational continuity. This component assesses not only the operator's physical risks but also considers cognitive workload and structural support within the work system [39,47]. In the baseline configuration used for the manual or semi-manual manufacturing case study, E comprises the Physical Ergonomic Indicator (PEI), Cognitive Ergonomic Indicator (CEI), and Macroergonomic Structure Indicator (MSI). However, the LSE–ORI framework does not require applying all ergonomic instruments in every manufacturing context. The choice of assessment must align with task characteristics, automation level, human-machine interaction patterns, and the dominant ergonomic risks. For instance, RULA and REBA need not be used concurrently if only one suits the activity's characteristics. Similarly, while NASA-TLX and MAS serve as the baseline configuration in this article, they may be replaced by other validated instruments if the manufacturing context requires a different cognitive or macroergonomic approach. This flexibility is governed by three conditions. First, the selected instruments must still represent the relevant ergonomic domains. Second, measurement results must be convertible to a 0–1 scale with a consistent interpretation—where higher values indicate ergonomic conditions that better support operational resilience. Third, document any reduction, substitution, or addition of instruments to ensure the E configuration remains traceable and replicable.

However, prior to conversion to the 0–1 scale, an indicator normalization process is required; specifically, positive normalization is applied, based on the principle that higher indicator values ​​signify better conditions:

(5) $${x_{i}}^{*}=\frac{x_{i\;}-\;x_{\min}}{x_{\max\;}-\;x_{\min}}$$

For indicators where higher values ​​signify worsening conditions, inverse normalization is used:

(6) $${x_{i}}^{*}=\frac{x_{\max}-\;x_{i}}{x_{\max\;}-\;x_{\min}}$$

where: ${x_{i}}^{*}$ = normalized indicator value; $x_{i}$ = actual indicator value; $x_{\min}$ = minimum value on the measurement scale; $x_{\max}$ = maximum value on the measurement scale. Thus, positive normalization is applied to benefit-type indicators, whereas inverse normalization is used for risk or burden indicators, such as RULA, REBA, and NASA-TLX.

When several normalized indicators need to be combined, the geometric mean is employed; this approach ensures that a high value in one indicator does not completely mask a low value in another [48,49] making it highly suitable for calculating the Physical Ergonomic Indicator. The concept of the geometric meaning is outlined below:

(7) $$\mathrm{Geometric}\;\mathrm{Mean}\;\left(\mathrm{GM}\right)=\prod_{i=1}^{n}zi^{1/n}$$

Where: **GM** = geometric mean aggregation value; $z_{i}$ = normalized value of the *i* ^th^ indicator; **n** = number of aggregated indicators.

### Physical ergonomic indicator

Physical ergonomic indicators are used to assess the extent to which the characteristics of physical activities support an operator's ability to maintain performance. The choice of instrument depends on the nature of the tasks and the type of work being observed. RULA is used for activities primarily involving the arms, wrists, neck, and upper body [34]. REBA is used for activities involving the whole body—such as bending, standing, reaching, dynamic postural changes, or manual material handling [33]. RULA and REBA may be used in conjunction if risks to both the upper body and the whole body are relevant. Other validated physical assessment instruments may be employed if the nature of the activity does not align with RULA or REBA criteria. Thus, the application of RULA and REBA is selective rather than mandatorily simultaneous.

RULA normalization is calculated using:

(8) $$\mathrm{RUL}A^{*}=\frac{\mathrm{RUL}A_{\max\;}-\;\mathrm{RULA}}{\mathrm{RUL}A_{\max\;}-\;\mathrm{RUL}A_{\min\;}}$$

With a RULA score range of 1–7:

(9) $$\mathrm{RULA}=\frac{7-\mathrm{RULA}}{6}$$

where: RULA^∗^ is the RULA value after inverse normalization; RULA is the actual RULA score; RULA_max_ is the maximum RULA score, which is 7; RULA_min_ is the minimum RULA score, which is 1. REBA normalization is calculated using:

(10) $$\mathrm{REB}A^{*}=\frac{\mathrm{REB}A_{\max\;}-\;\mathrm{REBA}}{\mathrm{REB}A_{\max\;}-\;\mathrm{REB}A_{\min\;}}$$

With a REBA score range of 1–15:

(11) $$\mathrm{REBA}=\frac{15-\mathrm{REBA}}{14}$$

Where: REBA^∗^ is the REBA value after inverse normalization; REBA is the actual REBA score; REBA_max_ is the maximum REBA score, which is 15; REBA_min_ is the minimum REBA score, which is 1.

If only RULA is used:

(12) $$\mathrm{PEI}=\mathrm{RUL}A^{*}$$

If only REBA is used:

(13) $$\mathrm{PEI}=\mathrm{REB}A^{*}$$

If multiple physical ergonomics tools are used simultaneously:

(14) $$\mathrm{PEI}={\left(\prod_{j=1}^{m}p_{j}^{*}\right)}^{1/m}$$

where: PEI= Physical Ergonomic Indicator; $P_{j}^{*}$= normalized value of the j^−th^ physical ergonomic indicator; m= number of physical ergonomic instruments used. Thus, RULA and REBA do not necessarily have to be used together; the choice between them depends on the nature of the physical activity.

### Cognitive ergonomic indicator

The Cognitive Ergonomic Indicator (CEI) is used to assess an operator's ability to handle mental demands, attention, time pressure, task complexity, and decision-making requirements [36]. Since higher NASA-TLX scores indicate a greater workload, an inverse normalization process is required to obtain relevant results, utilizing the following approach:

(15) $$\mathrm{CEI}=\mathrm{NAS}A^{*}=\frac{\mathrm{NAS}A_{\max\;}-\;\mathrm{NASA}}{\mathrm{NAS}A_{\max\;}-\;\mathrm{NAS}A_{\min\;}}$$

With a NASA-TLX range of 0–100:

(16) $$\mathrm{CEI}=\frac{100-\;\mathrm{NASA}\;\mathrm{TLX}}{100}$$

where: CEI is the Cognitive Ergonomic Indicator; $NASA^{*}$ is the NASA-TLX value after inverse normalization; NASA−TLX is the actual workload score; $NASA_{\text{max}}\;$is the maximum score (100); $NASA_{\text{min}}\;$min is the minimum score (0). NASA-TLX need not be used in every manufacturing context; other cognitive instruments may be employed if they better suit the nature of the task and the type of work being observed, provided they measure cognitive demands and their results can be normalized to the same scale.

### Macroergonomic structure indicator

The Macroergonomic Structure Indicator serves as a tool to assess the extent to which the work system structure supports the ability of operators and the organization to respond to operational disruptions, thereby contributing to the achievement of resilience within the work system [28,50]. In this configuration, the MSI is calculated using the Macroergonomic Analysis of Structure, which encompasses formalization, centralisation, and complexity [37,51]. The normalization of each dimension is calculated as follows:

(17) $$F^{*}=\frac{F-F_{\min}}{F_{\max}-\;F_{\min}}$$

(18) $$C^{*}=\frac{C-C_{\min}}{C_{\max}-\;C_{\min}}$$

(19) $$X^{*}=\frac{X-X_{\min}}{X_{\max}-\;X_{\min}}$$

where: ***F^∗^*** is the normalized formalization value, ***C*∗** is the normalized centralization value; ***X∗*** is the normalized complexity value; F, C, and X are the actual scores for each dimension; and the minimum and maximum indices indicate the limits of the measurement scale. The three dimensions are combined using:

(20) $$\mathrm{MSI}=\;{\left(F^{*}x\;C^{*}x\;X^{*}\right)}^{1/3}$$

where: **MSI** is the Macroergonomic Structure Indicator; $F^{*}$ represents the clarity of procedures and work standards; $C^{*}$represents the appropriateness of the distribution of authority; $X^{*}$ represents the manageability of communication, coordination, and inter-process relationships. The MAS instrument may be replaced by other macroergonomic methods if the organizational structure or work system within the manufacturing context is deemed to require a different instrument. However, any substitute indicator must still assess work system support, rather than merely the condition of individual operators.

### Calculation of the ergonomic resilience indicator

Once the PEI, CEI, and MSI have been obtained, the Ergonomic Resilience Indicator is calculated using the geometric mean concept:

(21) $$\mathrm{ERI}={\left(\mathrm{PEI}\;x\;\mathrm{CEI}\;x\;\mathrm{MSI}\right)}^{1/3}$$

ERI = ***Ergonomic Resilience Indicator value***; PEI= ***physical ergonomic capacity value***; CEI= ***cognitive ergonomics capacity value***; MSI= ***value of macroergonomic structural support***; $\frac{1}{3}$ = the rank used, because E is formed by three main subcomponents.

The use of the geometric mean preserves the contribution of all three dimensions and mitigates the risk of a high value in one aspect completely masking a low value in another [48,49]. The value of E ranges from 0 to 1. A value approaching 1 indicates that the operator's physical condition, cognitive workload, and work system structure collectively enhance operational resilience. Methodological flexibility is used to select physical instruments for the PEI. However, PEI, CEI, and MSI are retained as the three constituent domains of E to ensure the measurement does not reduce the human–system ergonomic capacity to a mere assessment of posture. Once the values ​​for L, S, and E are obtained, they can be utilized in the integration and vulnerability diagnosis stages described in the following subsection.

### Indicator integration and vulnerability diagnosis

Once the values ​​for the Lean Indicator (L), Six Sigma Indicator (S), and Ergonomic Resilience Indicator (E) have been obtained, these three components are integrated to produce the Operational Resilience Index (ORI). This integration occurs only after converting all indicators to a 0–1 scale with a consistent interpretation, where higher values indicate conditions increasingly conducive to operational resilience. Furthermore, the L, S, and E values ​​must be derived from the same unit of analysis and observation period to ensure that the measurement results consistently reflect the state of the production system. The ORI value can be calculated using the following multiplicative formula:

(22) ORI=L×S×E

where: ORI= ***Operational Resilience Index***, an index of operational resilience at the shop-floor level; L= ***Lean Indicator***, the value representing the smoothness of the process flow; S= ***Six Sigma Indicator***, the value representing quality stability; E= ***Ergonomic Resilience Indicator***, the value representing the ergonomic capacity of the human-system. Since L, S, and E are on a scale of 0–1, the ORI value also falls within the range:

(23) 0≤ORI≤1

An ORI value approaching 1 indicates that process flow smoothness, quality stability, and human-system ergonomic capacity collectively reinforce operational resilience. Conversely, a lower ORI value indicates weaknesses in one or more of its constituent components. Therefore, the ORI value should not be presented as a single figure without also reporting the L, S, and E values. A multiplicative formulation was selected because, in this method, operational resilience results from the simultaneous contribution of all three components [48,52]. A production system cannot be considered resilient if it merely exhibits smooth process flow while suffering from unstable quality or weak human-system ergonomic capacity. The multiplicative approach also mitigates the risk of a high value in one component masking a low value in another; thus, a weakness in any single component directly lowers the ORI value, helping to pinpoint the source of the production system's vulnerability.

The vulnerability diagnosis compares the L, S, and E values. The component with the lowest value is interpreted as the one providing the weakest support for operational resilience. If the L value is the lowest, the vulnerability relates primarily to the smooth flow of processes. If the S value is the lowest, the vulnerability relates primarily to quality stability. If the E value is the lowest, the vulnerability relates primarily to human-system ergonomic capacity. When E is the lowest component, the analysis compares the Physical Ergonomic Indicator (PEI), Cognitive Ergonomic Indicator (CEI), and Macroergonomic Structure Indicator (MSI). The lowest PEI value indicates that physical ergonomic conditions provide the weakest support. The lowest CEI value indicates that cognitive workload is the primary concern. Meanwhile, the lowest MSI value indicates that the work system structure—encompassing procedures, decision-making authority, communication, and coordination—is the weakest element. This diagnosis is evaluative in nature and does not establish a cause-and-effect relationship; the component with the lowest value merely identifies the part of the system requiring further examination and serves as the basis for prioritizing improvements.

Table 2 outlines the relationship between the lowest-scoring component and the necessary focus of evaluation. The initial diagnosis is conducted at the primary component level by comparing L, S, and E. This comparison indicates whether the weakest support for operational resilience stems from process flow, quality stability, or human-system ergonomic capacity. A second-level analysis is performed only if E is identified as the component with the lowest score. At this stage, the PEI, CEI, and MSI scores are compared to determine whether primary attention should focus on the operator's physical condition, cognitive workload, or work system structural support. This table does not reiterate the calculation procedures for each indicator, as the preceding subsection detailed the formulas for deriving L, S, PEI, CEI, MSI, and E. The diagnostic output does not provide a definitive causal conclusion; rather, it identifies the weakest component based on actual shop-floor conditions. These results serve to determine the evaluation focus and guide targeted improvements. The application of this diagnostic procedure is demonstrated in the Method Validation section using the SME case; consequently, this section focuses on component integration and interpretation.

**Table 2:** Guidance for diagnosing operational vulnerability based on the LSE–ORI components.

Diagnostic level	Component with the lowest value	Meaning within LSE–ORI	Data or aspects to examine	Evaluation direction	Method output
Main component	Lean Indicator (L)	Process flow provides the weakest contribution to operational resilience	Value-added time, total lead time, waiting time, transportation, queues, and non-value-added activities	Identify activities or process stages that restrict production flow	Process-flow vulnerability diagnosis
Main component	Six Sigma Indicator (S)	Quality stability provides the weakest contribution to operational resilience	Production volume, number of defects, defect opportunities, DPMO, actual sigma level, and rework	Identify product nonconformities and sources of quality instability	Quality-stability vulnerability diagnosis
Main component	Ergonomic Resilience Indicator (E)	Human–system ergonomic capacity provides the weakest contribution to operational resilience	PEI, CEI, and MSI values	Conduct further assessment of physical, cognitive, and macroergonomic components	Human–system ergonomic vulnerability diagnosis
Ergonomic subcomponent	Physical Ergonomic Indicator (PEI)	Physical working conditions provide the weakest contribution within the ergonomic component	RULA and/or REBA scores, posture, movement, load, frequency, and task characteristics	Identify tasks and postures associated with higher physical ergonomic risk	Physical ergonomic vulnerability diagnosis
Ergonomic subcomponent	Cognitive Ergonomic Indicator (CEI)	Operator cognitive capacity provides the weakest contribution within the ergonomic component	Mental demand, temporal demand, effort, performance, and frustration based on NASA-TLX	Identify sources of mental workload and task pressure	Cognitive ergonomic vulnerability diagnosis
Ergonomic subcomponent	Macroergonomic Structure Indicator (MSI)	Work-system structure provides the weakest contribution within the ergonomic component	Formalization, centralization, and complexity	Examine procedure clarity, decision authority, communication, and coordination	Macroergonomic vulnerability diagnosis

### Replication and adaptation procedure

Replicating the LSE–ORI method requires maintaining consistency in system boundaries, the unit of analysis, the observation period, and data sources. Researchers must clearly define the products or product groups being assessed, the start and end points, the workstations included in the measurement, the operators involved, and the data collection period. All process, quality, and ergonomics data must come from the same unit of analysis to ensure the L, S, E, and ORI values represent comparable production system conditions. Observation methods, definitions of value-added activities and defects, the number of defect opportunities, operator task characteristics, and macro-ergonomic assessment scales must also be documented to ensure the calculations are traceable and reproducible. Reapplication of the method must preserve five key elements. First, operational resilience remains shaped by smooth process flow, quality stability, and human-system ergonomic capacity. Second, these three constructs are represented by the Lean Indicator, Six Sigma Indicator, and Ergonomic Resilience Indicator. Third, all values ​​are converted to a 0–1 scale with a consistent interpretation: higher values ​​indicate conditions that better support operational resilience. Fourth, the ORI value continues to be calculated using the multiplicative formula ORI = L × S × E. Fifth, the ORI value must be reported alongside the L, S, and E values ​​so that the component providing the least support can be identified. These elements constitute the core structure of the method and remain unchanged when LSE–ORI is applied to different manufacturing subjects.

Methodological adjustments can be made to operational aspects of the implementation context—such as the unit of analysis, the number of workstations, the duration of the observation period, time-recording techniques, and sources of production data. The main flexibility in the ergonomics configuration lies in selecting physical assessment instruments. RULA is employed when activities predominantly involve the upper body, whereas REBA is used for activities involving whole-body posture, dynamic movements, or manual material handling. RULA and REBA may be used in tandem if both risk characteristics are relevant. However, cognitive ergonomics aspects (via NASA-TLX) and macroergonomics aspects (via MAS) are retained within the methodological configuration to ensure the Ergonomic Resilience Indicator is not reduced to a mere postural assessment.

The use of physical ergonomics instruments other than RULA and REBA may be considered in future research if the activity characteristics cannot be adequately represented by those two instruments. Such adjustments must be grounded in a clear methodology, yield scores convertible to a 0–1 scale, maintain a consistent interpretation direction, and be validated by relevant experts. As the demonstration in this article utilizes a configuration comprising RULA and/or REBA, NASA-TLX, and MAS, the use of other instruments is not claimed to have been validated through the same SME demonstration.

Replication also requires consistency in data processing. Formulas, instrument score thresholds, normalization methods, sigma targets, and aggregation procedures must be documented on the calculation sheet. If the sigma target or instrument configuration differs from the baseline demonstration, explicitly state the changes. ORI values ​​from two companies or manufacturing contexts should not be directly compared if they employ different system boundaries, defect definitions, instruments, or measurement configurations without a prior harmonization process. Subject to these conditions, LSE–ORI can be reapplied in manufacturing contexts possessing adequate process flow, quality, and ergonomics data, without restricting its use solely to the SME configuration. The manufacturing SME in this article serves as a demonstration of the method's application; generalizing it to other contexts requires adjustments that preserve the core construct while ensuring contextual validation.

## Method validation

The LSE–ORI method was evaluated through two complementary validation stages addressing different aspects of method quality. The first stage involved an SME shop-floor demonstration to examine whether the required process, quality, and ergonomics data could be collected within a consistent system boundary, transformed into standardized component scores, integrated into the composite index, and interpreted through the proposed diagnostic procedure. The second stage employed expert content validation to assess the relevance and conceptual appropriateness of the method architecture, indicator configuration, normalization and aggregation procedures, ORI formulation, and output interpretation. Together, these two stages were used to provide evidence of operational applicability, calculation traceability, and content validity. The validation was not intended to establish causal validity, intervention effectiveness, predictive performance, or longitudinal stability.

### SME demonstration

The applicability of the LSE–ORI method was demonstrated in a small and medium-sized manufacturing enterprise (SME) characterized by repetitive production processes and predominantly manual or semi-manual activities. The unit of analysis was a tractor spare-parts production line, with the system boundary extending from cutting and preparation to final inspection. Data were collected over a two-month observation period involving 13 operators. The same unit of analysis, system boundary, and observation period were applied consistently across the Lean, Six Sigma, and ergonomic components to ensure that the resulting L, S, E, and ORI values reflected the same operational conditions.

Process-flow data comprised value-added time and total lead time collected through direct observation and time measurement. Quality data were obtained from production and defect records, including production volume, defect counts, defect opportunities per unit, DPMO, and the resulting actual sigma level. Physical ergonomic conditions were assessed using RULA and/or REBA according to the characteristics of the observed activities, while cognitive workload was evaluated using NASA-TLX. Work-system structural support was assessed using the Macroergonomic Analysis of Structure (MAS), covering formalization, centralization, and complexity. The resulting measurements were then transformed, normalized, and aggregated according to the calculation procedures described in the Method details section. This demonstration therefore examined the operational applicability and calculation traceability of the LSE–ORI procedure within the observed SME context, rather than testing the effectiveness of an improvement intervention or establishing causal relationships.

The demonstration yielded L, S, PEI, CEI, MSI, E, and ORI values through the calculation sequence presented in Table 3 . The primary component scores were L = 0.60, S = 0.54, and E = 0.62, resulting in an ORI of approximately 0.20. Among the primary components, S scored lowest, indicating that quality stability provided the weakest support for operational resilience readiness in the demonstrated production line. Within the ergonomic component, MSI was the lowest-scoring subcomponent, indicating that work-system structural support required further examination. These results demonstrate how the method preserves component-level information while producing an integrated shop-floor readiness assessment.

**Table 3:** Worked calculation of the LSE–ORI method in the SME demonstration.

Component	Raw data	Calculation or transformation	Score	Output
Lean Indicator (L)	Value-added time and total lead time	Process Cycle Efficiency calculation	0.60	Process-flow capability
Six Sigma Indicator (S)	Production volume, defects, and defect opportunities	DPMO, actual sigma level, and normalization	0.54	Quality-stability capability
Physical Ergonomic Indicator (PEI)	RULA and/or REBA scores	Inverse normalization and physical score aggregation	0.73	Physical ergonomic capacity
Cognitive Ergonomic Indicator (CEI)	NASA-TLX score	Inverse normalization	0.65	Cognitive ergonomic capacity
Macroergonomic Structure Indicator (MSI)	Formalization, centralization, and complexity	Normalization and geometric mean	0.50	Work-system structural support
Ergonomic Resilience Indicator (E)	PEI, CEI, and MSI	Geometric mean	0.62	Human–system ergonomic capacity
Operational Resilience Index (ORI)	L, S, and E	ORI = L × S × E	0.20	Overall LSE–ORI result

### Expert content validation

Expert content validation was conducted to assess the relevance and conceptual appropriateness of the LSE–ORI method. The assessment covered the operational-resilience-readiness construct, selection of the L, S, PEI, CEI, and MSI indicators, normalization and aggregation procedures, ORI formulation, score interpretation, and weakest-component diagnostic logic. Fifteen experts with relevant academic and/or professional experience in manufacturing systems, Lean Six Sigma, quality engineering, ergonomics, performance measurement, and operational resilience participated in the assessment. The experts evaluated 64 items using a four-point relevance scale, with scores of 3 and 4 classified as relevant and scores of 1 and 2 indicating that an item required review.

Content validity was evaluated using the Item-level Content Validity Index (I-CVI), Scale-level Content Validity Index based on Average Agreement (S-CVI/Ave), and Scale-level Content Validity Index based on Universal Agreement (S-CVI/UA). Aiken’s V was additionally calculated to assess the strength of expert agreement, while modified kappa was used to account for agreement occurring by chance. These measures were applied consistently across the 64 assessment items to evaluate both item-level and overall method content validity. The authors also reviewed experts' qualitative comments to refine terminology, clarify potentially overlapping items, and improve the method's interpretation and implementation guidance.

All 64 items achieved an I-CVI value of at least 0.933. The S-CVI/Ave value of 0.995 and the S-CVI/UA value of 0.922 indicate a very high level of agreement on the method's overall content. The average Aiken’s V of 0.946 and average modified kappa of 0.995 provide further evidence of strong content validity. Fifty-nine items achieved universal agreement, whereas the remaining five items received only one rating below their respective categories. Given the consistently high I-CVI values across all items, no items were removed. The five items without universal agreement were retained after minor revisions to improve wording clarity and reduce potential interpretive overlap. Table 4 presents the detailed results. These findings support the content validity of the LSE–ORI method but do not establish its causal validity, sensitivity to operational changes, intervention effectiveness, or longitudinal stability.

**Table 4:** Summary of expert content-validity assessment of the LSE–ORI method.

Validation indicator	Assessment basis	Result	Interpretation
Number of experts	Experts with relevant academic and/or professional experience	15	Adequate expert representation
Number of assessed items	Items covering the construct, indicators, Normalization, formulation, interpretation, and vulnerability diagnosis	64	Comprehensive method assessment
Mean expert rating	Four-point relevance scale	3.838/4.000	Very high overall relevance
Minimum I-CVI	Proportion of experts assigning a relevance score of 3 or 4 to each item	0.933	High item-level content validity
I-CVI range	Item-level content-validity results	0.933–1.000	High to universal expert agreement
S-CVI/Ave	Average of all I-CVI values	0.995	Excellent overall content validity
S-CVI/UA	Proportion of items receiving universal agreement	0.922	Very high universal agreement
Aiken’s V	Strength of expert agreement across the four-point rating scale	0.946	Strong content validity
Mean modified kappa	Chance-corrected expert agreement	0.995	Excellent agreement beyond chance
Universally agreed items	Items rated relevant by all experts	59 of 64	Most items received complete agreement
Items requiring minor review	Items receiving one rating below the relevant category	5 of 64	Minor wording review; no item deletion required

Taken together, the SME demonstration and expert content validation provide complementary evidence for the current stage of LSE–ORI method development. The SME demonstration showed that the required shop-floor data could be processed through the proposed calculation sequence and retained as traceable component-level diagnostic information, while the expert assessment supported the relevance and conceptual appropriateness of the method architecture and calculation logic. These findings support the operational applicability, calculation traceability, and content validity of the proposed method within the scope examined. Further studies are required to evaluate measurement repeatability, sensitivity to changing operational conditions, and transferability across different manufacturing settings.

## Conclusion

The LSE–ORI method provides a structured and non-interventional approach for assessing shop-floor operational resilience readiness by integrating process-flow efficiency, quality stability, and human–system ergonomic capacity within a common 0–1 measurement framework. In the SME demonstration, the Lean Indicator (L), Six Sigma Indicator (S), and Ergonomic Resilience Indicator (E) yielded scores of 0.60, 0.54, and 0.62, respectively, resulting in an ORI of approximately 0.20. The Six Sigma Indicator was the lowest-scoring primary component, while the Macroergonomic Structure Indicator (MSI) was the lowest ergonomic subcomponent. Expert content validation involving 15 experts and 64 items yielded a minimum I-CVI of 0.933 and an S-CVI/Ave of 0.995. These findings support the operational applicability, calculation traceability, and content validity of the method within the demonstrated context. LSE–ORI should be interpreted as a readiness-assessment and diagnostic method rather than as a causal or predictive measure of resilience. Further evaluation is required to establish repeatability, sensitivity, and transferability across different manufacturing settings.

## Limitation

The LSE–ORI method has several limitations. The demonstration was conducted within a single manufacturing-based small and medium-sized enterprise (SME); therefore, applying it to manufacturing processes with different characteristics will require adaptation and further testing. The reliability of the index values ​​depends on the completeness, accuracy, and consistency of shop-floor data. RULA and REBA assessments depend on observation accuracy, whereas NASA-TLX and MAS rely on participant perceptions and responses. Comparing ORI values across companies is feasible only if system boundaries, units of analysis, indicator definitions, observation periods, instruments, and normalization procedures are comparable. Furthermore, while expert validation in this study provides evidence of content validity, it does not yet demonstrate intervention effectiveness, cause-and-effect relationships, index sensitivity to changing conditions, or longitudinal stability of results across diverse manufacturing contexts.

## Declaration of generative AI use

During the preparation of this work, the authors used ChatGPT (OpenAI) to improve the clarity and organization of the manuscript. After using this tool, the authors reviewed and edited the content as needed and take full responsibility for the published article.

## Ethics statements

This study received ethical approval from the Research Ethics Committee for Systems and Industrial Engineering at the Faculty of Engineering, Universitas Maarif Hasyim Latif, via Ethical Clearance Letter Number 0041/KE/FT-UMAHA/X/2025. Issued in Sidoarjo on October 24, 2025, the approval is valid from October 25, 2025, to October 25, 2026. The protocol underwent an expedited review process and was classified as minimal-risk research. The study involved observations of shop-floor activity, questionnaire-based assessments, and expert interviews. All participants were informed of the study's objectives, procedures, the voluntary nature of participation, and data usage, and provided written consent prior to participating. Participant identities and organizational information have been kept confidential, and only aggregated or anonymized data are presented in this article.

## CRediT authorship contribution statement

**Deri Maryadi:** Conceptualization, Methodology, Validation, Formal analysis, Investigation, Data curation, Visualization, Writing – original draft. **Moses Laksono Singgih:** Conceptualization, Methodology, Supervision, Writing – review & editing. **Dyah Santhi Dewi:** Conceptualization, Methodology, Validation, Supervision, Writing – review & editing.

## Declaration of interests

The authors declare that they have no known competing financial interests or personal relationships that could have appeared to influence the work reported in this paper.

## Data Availability

Data will be made available on request.

## Appendix Supplementary materials

**Supplementary material** A supplementary Excel workbook is provided containing the data-collection template, anonymized SME demonstration data, formula-based LSE–ORI calculations, the final expert content-validation instrument, anonymized expert ratings, and the corresponding content-validity analyses. Additional company records are not publicly available because of confidentiality restrictions.

## References

[1] saad M.H., Hagelaar G., van der Velde G., Omta S.W.F. (2021). Conceptualization of SMEs’ business resilience: a systematic literature review. Cogent Bus. Manag..

[2] Maryadi D., Singgih M.L., Dewi D.S. (2025). 2025 9th International Conference on Information Technology, Information Systems and Electrical Engineering.

[3] Shah R., Ward P.T. (2007). Defining and developing measures of lean production. J. Oper. Manag..

[4] Maryadi D. (2021). Lean six sigma DMAIC implementation to reduce total lead time internal supply chain process. https://ieomsociety.org/proceedings/2021indonesia/394.pdf.

[5] Maryadi D., Moulita R.N., King M.L., Veranika R.M., Madagaskar M. (2024). Value stream mapping for warehouse process in automotive manufacturing case. Int. J. Mech. Comput. Manuf. Res..

[6] Condé G.C.P., Oprime P.C., Pimenta M.L., Sordan J.E., Bueno C.R. (2023). Defect reduction using DMAIC and Lean Six Sigma: a case study in a manufacturing car parts supplier. Int. J. Qual. Reliab. Manag..

[7] Sreekanth V., Kavilal E.G., Krishna S., Mohan N. (2024). Implementation of six Sigma methodology in a medical equipment manufacturing company. TQM J..

[8] Adiyanto O., Mohamad E., Jaafar R., Ma F., Faishal M., Anggraeni A. (2022). Application of Nordic body map and rapid upper limb assessment for assessing work-related musculoskeletal disorders: a case study in small and medium enterprises. Int. J. Integr. Eng..

[9] Vandergrift J.L., Gold J.E., Hanlon A., Punnett L. (2012). Physical and psychosocial ergonomic risk factors for low back pain in automobile manufacturing workers. Occup. Environ. Med..

[10] B. Poduval, Q. Lu, J. Guo, S. Ding, and S. Zhang, “Workload analysis using Modified Full Time Equivalent (M-FTE) and NASA-TLX methods to optimize engineer headcount in the engineering services department Workload analysis using Modified Full Time Equivalent (M- FTE) and NASA-TLX methods to optimize en”, doi: 10.1088/1757-899X/1072/1/012036.

[11] Javernik A., Buchmeister B., Ojstersek R. (2023). the Nasa-Tlx approach to understand workers workload in Human-robot collaboration. Int. J. Simul. Model..

[12] Hammad M., Islam M.S., Salam M.A., Jafry A.T., Ali I., Khan W.A. (2023). Framework for the implementation of smart manufacturing systems: a case in point. Processes.

[13] Ariyanti F.D., Putri A.C., Ningtyas D.A. (2021). Implementation of lean construction and critical chain project management (CCPM) for waste management and work estimation on the Ciawi dam construction project. IOP Conf. Ser. Earth Environ. Sci..

[14] Vashishth A., Lameijer B.A., Chakraborty A., Antony J., Moormann J. (2024). Implementing Lean Six Sigma in financial services: the effect of motivations, selected methods and challenges on LSS program- and organizational performance. Int. J. Qual. Reliab. Manag..

[15] Ababou M., Chelh S., Elhiri M. (2023). A bibliometric analysis of the literature on food industry supply chain resilience: investigating key contributors and global trends. Sustainability.

[16] Fowler D.S., Epiphaniou G., Higgins M.D. (2023). Aspects of resilience for smart manufacturing systems,. Strateg. Change.

[17] Zarrin M. (2016). Simulation optimization of lean production strategy by considering resilience engineering in a production system with maintenance policies. SIMUL-T. SOC. MOD. SIM..

[18] Khalfallah M., Lakhal L. (2021). The impact of lean manufacturing practices on operational and financial performance: the mediating role of agile manufacturing. Int. J. Qual. Reliab. Manag..

[19] Sari A.D., Hardiansa F., Suryoputro M.R. (2018). Workload assessment on foundry SME to enhance productivity using full time equivalent. MATEC Web Conf..

[20] Ambrogio G., Filice L., Longo F., Padovano A. (2022). Workforce and supply chain disruption as a digital and technological innovation opportunity for resilient manufacturing systems in the COVID-19 pandemic. Comput. Ind. Eng..

[21] Afonso M., Teresa A., Godina R. (2022). Proposal of an innovative ergonomic SMED model in an automotive steel springs industrial unit. Adv. Ind. Manuf. Eng..

[22] Tortorella G.L., Garcia L., Vergara L., Ferreira E.P. (2017). Lean manufacturing implementation : an assessment method with regards to socio-technical and ergonomics practices adoption. Int. J. Adv. Manuf. Technol..

[23] Nagaraj T.S., Jeyapaul R. (2021). The Management of Operations an empirical investigation on association between human factors, ergonomics and lean manufacturing. Prod. Plan. Control.

[24] Aruväli T., De Marchi M., Rauch E. (2023). Analysis of quantitative metrics for assessing resilience of human - centered CPPS workstations. Sci. Rep..

[25] Dittfeld H., Van Donk D.P., Van Huet S. (2022). The effect of production system characteristics on resilience capabilities : a multiple case study. Int. J. Oper. Prod. Manag..

[26] Trieu H.D.X., Van Nguyen P., Nguyen T.T.M., Vu H.T.M., Tran K.T. (2023). Information technology capabilities and organizational ambidexterity facilitating organizational resilience and firm performance of SMEs. Asia Pacific Manag. Rev..

[27] Azevedo S.G., Carvalho H., Cruz-Machado V. (2016). LARG index: a benchmarking tool for improving the leanness, agility, resilience and greenness of the automotive supply chain. Benchmarking.

[28] Taghi-Molla A., Rabbani M., Karimi Gavareshki M.H., Dehghani E. (2020). Safety improvement in a gas refinery based on resilience engineering and macro-ergonomics indicators: a Bayesian network–artificial neural network approach. Int. J. Syst. Assur. Eng. Manag..

[29] Gaël M., René A., Christine C. (2009). How good micro /macro ergonomics may improve resilience, but not necessarily safety. Saf. Sci..

[30] Mazziotta M., Pareto A. (2016). On a generalized non-compensatory composite index for measuring socio-economic phenomena. Soc. Indic. Res..

[31] M. Nardo, M. Saisana, A. Saltelli, S. Tarantola, A. Hoffmann, and E. Giovannini, “Handbook on constructing composite indicators : methodology and user guide,” 2005.

[32] Greco S., Ishizaka A., Tasiou M., Torrisi G. (2019). On the methodological framework of composite indices : a review of the issues of weighting, aggregation, and robustness. Soc. Indic. Res..

[33] McAtamney L., Hignett S., McAtamney L., Hignett S. (2004). Handbook of Human Factors and Ergonomics Methods.

[34] L. Mcatamney and E.N. Corlett, “RULA : a survey method for the investigation of work-related upper limb disorders,” vol. 24, no. 2, pp. 91–99, 1993. 10.1016/0003-6870(93)90080-s 15676903

[35] Lund S. (2021). NASA-TLX assessment of workload in resident physicians and faculty surgeons covering trauma, surgical intensive care unit, and emergency general surgery services. Am. J. Surg..

[36] Babaei E., Dingler T., Tag B., Velloso E. (2025). Should we use the NASA-TLX in HCI? A review of theoretical and methodological issues around mental workload measurement. Int. J. Hum. Comput. Stud..

[37] Ong A.K.S., Cai D., Castillo S., Chang S.Q., Valenton G., Mejia E.J.B. (2025). Macroergonomic factors, coping style, and satisfaction analysis for nursing career pursuance: a perspective from the Philippines. Acta Psychol. (Amst)..

[38] A. Azadeh, E. Roudi, and V. Salehi, “Optimum safety design approach based on integrated macro- ergonomics and resilience engineering in a tile and ceramic factory optimum design approach based on integrated macro-ergonomics and resilience engineering in a tile and ceramic factory,” pp. 0–33, 2017.

[39] Azadeh A., Zarrin M. (2016). An intelligent framework for productivity assessment and analysis of human resource from resilience engineering, motivational factors, HSE and ergonomics perspectives. Saf. Sci..

[40] M. Hossain and M.K. Uddin, “An approach to improve the process cycle efficiency and reduce the lead time of a mango juice processing line by using lean tools: a case study,” vol. 6, no. 1, pp. 1442–1452, 2015.

[41] Daniyan I., Adeodu A., Mpofu K., Maladzhi R., Grace M., Katumba K. (2022). Heliyon application of lean six Sigma methodology using DMAIC approach for the improvement of bogie assembly process in the railcar industry. Heliyon.

[42] B.M. Kleiner, “Macroegonomics : work system analysis and design,” vol. 50, no. 3, pp. 461–467, 2008, doi: 10.1518/001872008×288501. 10.1518/001872008X288501 18689054

[43] Antony J., Snee R., Hoerl R. (2017). Lean Six Sigma: yesterday, today and tomorrow. Int. J. Qual. Reliab. Manag..

[44] L.M. Syikilili and M.L. Singgih, “A systematic review of Lean Six Sigma and HACCP integration in the food industry : toward manufacturing excellence and compliance,” 13, pp. 50–58, 2026, doi: 10.18502/jfqhc.13.1.21380.

[45] Drohomeretski E., Gouvea Da Costa S.E., Pinheiro De Lima E., Garbuio P.A.D.R. (2014). Lean, six sigma and lean six sigma: an analysis based on operations strategy. Int. J. Prod. Res..

[46] Srinivasan K., Muthu S., Devadasan S.R., Sugumaran C. (2016). Six Sigma through DMAIC phases : a literature review,. Int. J. Product. Qual. Manag..

[47] Azadeh A., Roudi E., Salehi V. (2017). Optimum design approach based on integrated macro-ergonomics and resilience engineering in a tile and ceramic factory. Saf. Sci..

[48] F. Mariani and M. Ciommi, “Aggregating composite indicators through the geometric mean : a penalization approach,” 2022.

[49] Dalton K.V, Greenlees J.S., Stewart K.J. (1998). Incorporating a geometric mean formula into the CPI. Mon. Labor Rev..

[50] Habibifar N., Hamid M., Nisiri M.M. (2019). Concurrent optimization of integrated macro-ergonomics and resilience engineering in a pharmaceutical manufacturer. Int. J. Ind. Syst. Eng..

[51] Realyvásquez A., Maldonado-Macías A.A., García-Alcaraz J.L., Blanco-Fernández J. (2015). Effects of organizational macroergonomic compatibility elements over manufacturing systems’ performance. Proc. Manuf..

[52] Zhou P., Ang B.W., Zhou D.Q. (2010). Weighting and aggregation in composite indicator construction: a multiplicative optimization approach. Soc. Indic. Res..

